# Practical Benefits of Single- vs. Three-Port Laparoscopic Appendectomy for Pain Relief and Long-Term Cosmesis in Pediatric Patients: A Prospective Comparative Study

**DOI:** 10.3390/jcm14197077

**Published:** 2025-10-07

**Authors:** Tae Ah Kim, Won Me Kang, Soo Min Ahn

**Affiliations:** 1 Department of Surgery, Asan Medical Center, Ulsan University College of Medicine, Seoul 05505, Republic of Korea; white4829@gmail.com; 2 Department of Surgery, Hallym University Sacred Heart Hospital, Anyang 14068, Republic of Korea; bbome@hallym.ac.kr; 3 Department of Pediatric Surgery, Gangnam Severance Hospital, Yonsei University College of Medicine, Seoul 06273, Republic of Korea

**Keywords:** single-port laparoscopic appendectomy, pediatric patient, postoperative pain, cosmetic satisfaction, minimal clinically important difference, propensity score matching

## Abstract

**Background/Objectives**: Comparative studies examining postoperative pain and cosmetic outcomes following single-port laparoscopic appendectomy (SLA) and three-port laparoscopic appendectomy (TLA) in pediatric patients with appendicitis have produced inconsistent results. We aimed to determine whether SLA offers practical benefits over TLA in terms of recovery-phase pain relief and long-term cosmetic satisfaction in pediatric patients. **Methods**: This prospective comparative study included children aged 15 years or younger who underwent laparoscopic appendectomy for uncomplicated acute appendicitis. The degree of pain reduction was compared between the SLA and TLA groups on postoperative days (PODs) 1, 2, and 7, both at rest and during coughing and ambulation, using the Visual Analog Scale for Pain (VASP). Global cosmetic satisfaction was assessed at 1 month and 3 years postoperatively using the Visual Analog Scale for Cosmesis (VASC). Scar perception was evaluated with the Patient and Parental Scar Assessment Scale (PSAS). The primary outcome was the degree of pain reduction during ambulation on POD7. The secondary outcome was global cosmetic satisfaction at 3 years. Propensity score matching (PSM) was used as a sensitivity analysis to control for baseline differences. Continuous variables were assessed for normality using the Shapiro–Wilk test. **Results**: Baseline characteristics were similar among 238 patients (127 SLA and 111 TLA). SLA resulted in significantly greater pain reduction during ambulation on POD7 (deltaVASP7_walk: −6.22 ± 2.60 vs. −5.06 ± 3.23, *p* < 0.01, mean difference = −1.16, Cohen’s d = 0.39). However, this difference did not reach the minimal clinically important difference (MCID) threshold of 1.3. PSM analysis with 82 matched pairs confirmed the results, with even larger effect sizes. At 3 years, the SLA group reported significantly higher cosmetic satisfaction (VASC: median 10 [9–10] vs. 8 [6–9], *p* < 0.001, r = 0.44), surpassing the MCID of 1.5. The TLA group scored worse in scar perception regarding color, stiffness, thickness, and irregularity. Mediation analysis indicated that 66% of the overall effect on cosmetic satisfaction was mediated by scar perception. **Conclusions**: Although SLA offers statistically significant yet clinically marginal benefits in early postoperative pain reduction, it provides substantial benefits in long-term cosmetic satisfaction compared with TLA in pediatric patients.

## 1. Introduction

Acute appendicitis is the most common cause of emergency abdominal surgery among children, impacting approximately 70,000 pediatric patients annually in the United States [1]. The surgical management of pediatric appendicitis has evolved considerably over the past twenty years, with laparoscopic appendectomy becoming the preferred method due to its well-documented benefits, including diminished postoperative pain, abbreviated hospital stays, decreased rates of wound infection, significantly fewer intraabdominal adhesions, and faster resumption of normal activities, compared with open surgical procedures [2,3,4,5]. As advancements in minimally invasive techniques progressed, single-port laparoscopic appendectomy (SLA) was introduced as a potential improvement to traditional three-port laparoscopic appendectomy (TLA). SLA involves the utilization of a single transumbilical incision, which can be concealed within the natural contour of the umbilicus, offering potential functional and esthetic benefits [6]. These advantages encompass reduced parietal trauma due to fewer incisions, decreased postoperative pain, and improved cosmetic results through scar concealment [7]. Recent systematic reviews and meta-analyses focusing on adult populations have affirmed that SLA is a safe and feasible procedure, with operative durations, conversion rates, and complication profiles comparable to those of TLA [8,9].

Nevertheless, the practical advantages of SLA in pediatric populations are debated, as studies present conflicting outcomes. While some investigations have reported notable pain alleviation with SLA, indicating mean pain scores of 3.93 at 12 h postoperatively compared with 5.32 with the traditional laparoscopic approach [10], others have observed no significant differences in pain metrics or analgesic consumption between the two techniques [11,12]. A recent multicenter study conducted in China, involving experienced pediatric surgeons, found no significant differences in operative duration, early complications, or postoperative pain between SLA and TLA. Nonetheless, the study noted superior long-term cosmetic satisfaction following SLA [13]. Likewise, a meta-analysis of pediatric research yielded heterogeneous findings, with some studies demonstrating reduced pain and others indicating equivalence, underscoring the need for meticulously designed prospective research to establish definitive conclusions [14].

Evaluating cosmetic outcomes presents additional challenges. Both parents and pediatric patients are sensitive to surgical scarring, which may influence psychological well-being and self-esteem during critical developmental stages [15]. However, most prior research has relied on short-term assessments, typically conducted 1–6 months postoperatively, which may overlook important long-term variations. Since scars continue to remodel and mature over 12–24 months, delayed assessments are essential to capturing the full trajectory of cosmetic outcomes [16]. Moreover, most studies utilized non-validated or surgeon-reported esthetic scales rather than patient-reported outcome measures, thereby constraining the interpretative scope of their findings [17].

Notably, the existing literature has several methodological limitations that restrict its clinical relevance. First, most studies do not incorporate the concept of the minimal clinically important difference (MCID), making it challenging to distinguish between statistical and clinical significance [18]. Second, the lack of activity-specific pain assessments (such as pain while resting, coughing, and walking) may overlook essential functional differences between techniques [19]. Third, the absence of a detailed analysis of scar characteristics precludes an understanding of which specific scar features influence patient satisfaction [20]. Finally, few studies have used appropriate statistical methods to account for confounding factors in non-randomized comparisons [21].

In this study, we address these limitations by adopting a prospective comparative design with several methodological strengths: (1) the inclusion of established MCID thresholds for both pain (1.3 points) and cosmetic satisfaction (1.5 points) based on validated pediatric studies [22,23]; (2) activity-specific pain assessment at multiple time points to capture recovery patterns; (3) long-term follow-up at 3 years with validated patient-reported outcome measures, including the Visual Analog Scale for Cosmesis (VASC) and the six-item Patient and Observer Scar Assessment Scale (PSAS) [24,25]; (4) propensity score matching to control for baseline confounding factors; (5) mediation analysis to explore the mechanistic pathway from surgical technique to patient satisfaction. We hypothesized that SLA offers better pain control during early recovery and improved long-term cosmetic satisfaction compared with TLA in pediatric patients with uncomplicated appendicitis.

## 2. Materials and Methods

### 2.1. Study Design and Population

This prospective comparative study was conducted at Hallym University Sacred Heart Hospital from April 2016 to August 2021. The study protocol was approved by the Institutional Review Board (IRB 2014-I124, approval date: 31 August 2016) and registered at clinicaltrials.gov (Identifier: NCT03106467). Written informed consent was obtained from the parents or legal guardians of all participants. The study population consisted of children aged 15 years or younger who underwent laparoscopic appendectomy for uncomplicated acute appendicitis. Acute appendicitis was diagnosed based on clinical, laboratory, and radiographic evaluation (abdominoperineal CT scan or ultrasonography), including history of right lower quadrant pain, presence of right lower quadrant tenderness on physical examination, fever > 38 °C, and/or white blood cell count > 10 × 10^3^ cells/mL. Exclusion criteria included radiologically and surgically confirmed panperitonitis or periappendiceal abscess requiring drainage catheter placement, previous abdominal surgery, ≥3 days of empirical antibiotic therapy before diagnosis, history of coagulation disorders, contraindication to general anesthesia, suspected or proven malignancy, and mental illness.

**Figure 1:**
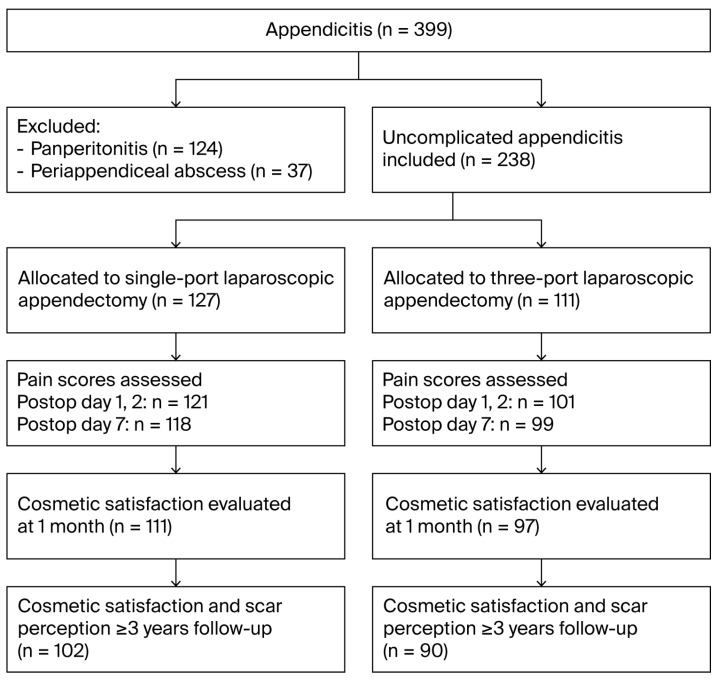
STROBE flow diagram of patient selection and follow-up.

A total of 399 pediatric patients with suspected appendicitis were assessed for eligibility. After excluding 161 patients with complicated appendicitis, 238 patients with uncomplicated appendicitis were included: 127 underwent SLA, and 111 underwent TLA (Figure 1). Pain data were collected on postoperative days 1, 2, and 7. Cosmetic satisfaction (VASC) was assessed at 1 month and at 3 years or later. Scar perception was also evaluated at the ≥3-year follow-up using the six-item validated Scar Assessment Questionnaire (Patient and Parental Scar Assessment Scale, PSAS) (Table A1).

### 2.2. Surgical Techniques

All procedures were performed by two fellowship-trained pediatric surgeons with experience of more than 100 cases each of both SLA and TLA prior to the initiation of this study. Every operation was recorded for quality control and periodic review.

Single-port laparoscopic appendectomy: A 1.5 cm curvilinear skin incision was made within the umbilicus, with a 2.0 cm umbilical fascial opening. A commercially available single-port device (Glove Port; Inframed Meditech, Seoul, Republic of Korea) was introduced. After establishing pneumoperitoneum at 8 mmHg, diagnostic laparoscopy was performed using a 5 mm, 30-degree rigid laparoscope. The Appendix was manipulated using roticular angulated instruments (Covidien, Mansfield, MA, USA) and conventional straight laparoscopic instruments (Ethicon, Cincinnati, OH, USA). The mesoappendix was dissected using electrocautery, and periappendiceal vessels were ligated with Hem-O-lock clips (Weck; Teleflex, Wayne, PA, USA). The Appendix was then ligated using polydioxanone loops (Ethicon, Cincinnati, OH, USA). The specimen was retrieved via a transumbilical port with a retrieval pouch (LapBag; Sejong Medical, Republic of Korea).

Three-port laparoscopic appendectomy: TLA employed a 5 mm or 10 mm 30-degree rigid scope through a 0.5 to 1.0 cm intraumbilical incision, with two additional 5 mm incisions in the left lower quadrant and the suprapubic area. Dissection and ligation techniques were identical to those used in the SLA procedure. Specimen retrieval was performed through the intraumbilical incision, using the same retrieval pouch system.

### 2.3. Postoperative Care and Follow-Up

All patients received standardized antibiotic therapy (30 mg/kg of cefuroxime every 8 h, with a maximum dose of 750 mg) from diagnosis through postoperative day 2. Standardized analgesic protocol included a single fentanyl citrate injection (1 mcg/kg) before awakening from anesthesia and ketorolac tromethamine (0.5 mg/kg) in the recovery room. Additional analgesics were administered for VASP scores of 6 or higher, with a minimum 4 h interval between doses. The patients were discharged when they were able to tolerate a regular diet, typically on postoperative day 2, and followed up with 1 week postoperatively at the outpatient clinic.

### 2.4. Outcome Measurements

Primary Outcome: The primary outcome was the magnitude of pain reduction during walking on postoperative day 7 (deltaVASP7_walk), calculated as the difference between preoperative and postoperative pain scores. The co-primary outcome was pain reduction during walking on postoperative day 2 (deltaVASP2_walk). Pain assessments utilized the Visual Analog Scale for Pain (VASP, 0–10 scale), measured preoperatively and on postoperative days 1, 2, and 7 [22]. Pain was evaluated during three activities: rest, coughing (three intentional coughs), and ambulation (10-step marching in place). The MCID for VASP was established at 1.3 based on the pediatric literature [22].

Secondary Outcome: The secondary outcome was global cosmetic satisfaction, assessed using the Visual Analog Scale for Cosmesis (VASC, 0–10 scale), at 1 month and 3 years postoperatively [23]. The MCID for cosmetic satisfaction was set at 1.5 [23].

Scar perception was evaluated at 3 years using the validated Patient and Parental Scar Assessment Scale (PSAS), which assesses six items: pain, itchiness, color, stiffness, thickness, and irregularity (each scored 0–10, with 10 representing the worst imaginable outcome) [24,25].

### 2.5. Sample Size Calculation

For the primary outcome, assuming a pain reduction MCID of 1.3, standard deviation of 3.2, power of 80%, and 10% dropout rate, 106 patients per group were required. For the secondary outcome, assuming a cosmetic satisfaction MCID of 1.5, standard deviation of 2.0, power of 80%, and 20% dropout rate at 3 years, 35 patients per group were required.

### 2.6. Statistical Analysis

Continuous variables were first assessed for normality using the Shapiro–Wilk test, with additional evaluations of skewness and kurtosis. Variables were considered normally distributed if they met the criteria of |skewness| < 1 and |kurtosis| < 3. Non-normally distributed variables are expressed as median [interquartile range] values and were compared using the Mann–Whitney U test. Effect sizes were calculated using Cohen’s d for normally distributed variables and r (r = Z/√N) for non-normally distributed variables. The magnitude of effect sizes was interpreted as small (d = 0.2, r = 0.1), medium (d = 0.5, r = 0.3), or large (d = 0.8, r = 0.5). Statistical significance was set at *p* < 0.01. Statistical analyses were performed using the Statistical Package for Social Sciences (SPSS, Version 27.0, IBM Corp., Armonk, NY, USA).

To address potential selection bias inherent in the non-randomized design, propensity score matching (PSM) was performed as a sensitivity analysis. Propensity scores were estimated using a logistic regression model including baseline covariates: age, sex, weight, BMI, white blood cell count, neutrophil percentage, and CRP. Due to the non-normal distribution of BMI, neutrophil percentage, and CRP, these variables were log-transformed before their inclusion in the propensity score model. For the propensity score matched analysis, paired *t*-tests were used for normally distributed variables, and Wilcoxon signed-rank tests were used for non-normally distributed variables. Nearest-neighbor matching with a caliper of 0.10 was used to create matched pairs. The quality of matching was assessed by comparing standardized mean differences before and after matching, with differences < 0.1 indicating good balance.

Causal mediation analysis was performed to evaluate the relationship between surgical technique, scar perception, and cosmetic satisfaction using bias-corrected bootstrapping (5000 resamples). The analysis decomposed the total effect into the Average Causal Mediation Effect (ACME), representing the indirect effect mediated through scar perception, and the Average Direct Effect (ADE), representing the direct effect not mediated through scar perception.

## 3. Results

### 3.1. Patient Characteristics and Surgical Outcomes

Baseline characteristics were comparable between groups (Table 1). The mean age was 10.1 ± 3.0 years in the SLA group and 9.8 ± 3.1 years in the TLA group (*p* = 0.407). Gender distribution, weight, BMI, laboratory parameters (WBC, neutrophil percentage, and CRP), operation time, and postoperative length of stay showed no significant differences between groups. Of the 238 patients enrolled, 208 (87.4%) completed the 1-month follow-up and 192 (80.7%) completed the 3-year follow-up. The follow-up rates were similar between groups (SLA: 80.3% vs. TLA: 81.1% at 3 years, *p* = 0.877), suggesting minimal attrition bias. There was no conversion to laparotomy or adverse events during the operation in either group. Postoperative seroma collection and ileus were managed conservatively.

### 3.2. Pain Outcomes

**Figure 2:**
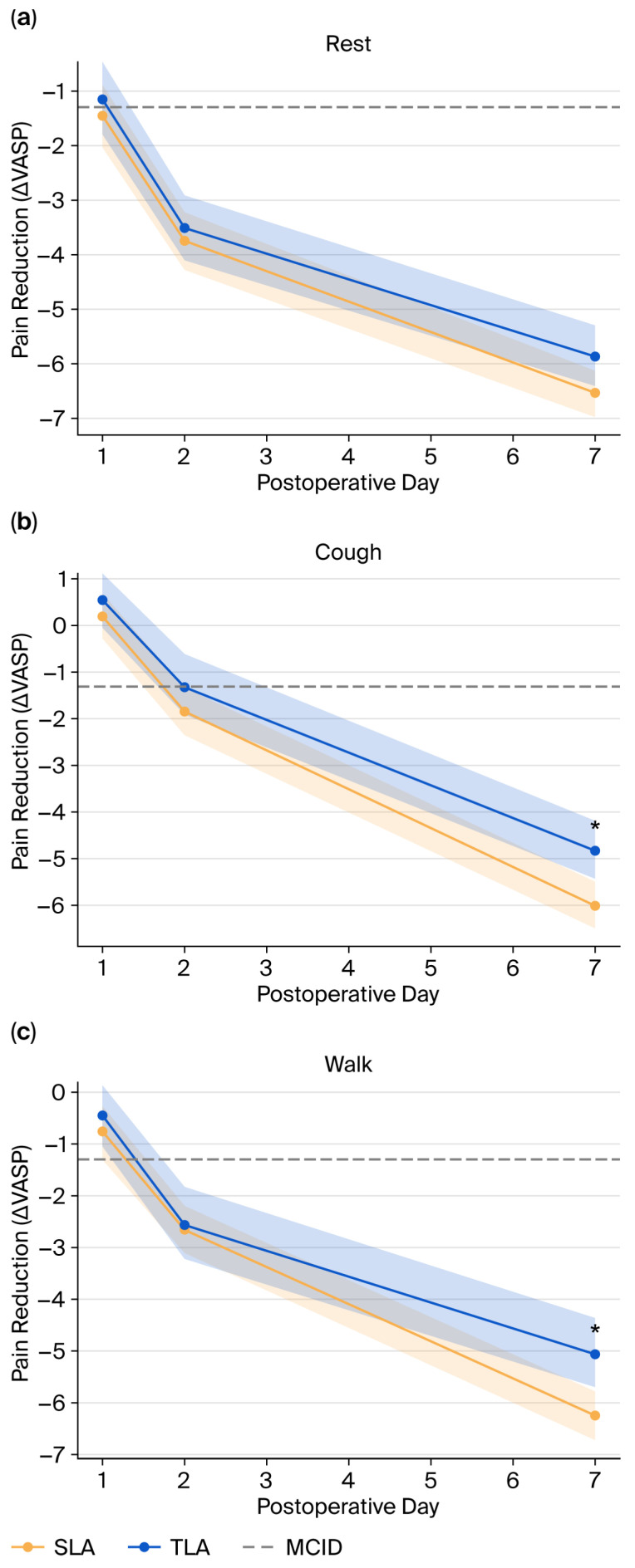
Postoperative pain reduction over time in SLA and TLA groups. Line plots represent changes in Visual Analog Scale scores for pain from baseline at rest (**a**), during coughing (**b**), and walking (**c**) on postoperative days (PODs) 1, 2, and 7 (deltaVASPn_pain, cough, or walk). ΔVASP was calculated as the difference from baseline. Shaded bands indicate 95% confidence intervals. The minimal clinically important difference (MCID) threshold of 1.3 is indicated by the dashed horizontal line. SLA (single-port laparoscopic appendectomy) is shown in orange; TLA (three-port laparoscopic appendectomy) is shown in blue. Asterisks (*) denote statistically significant between-group differences at POD7 (*p* < 0.01).

Pain reduction outcomes are summarized in Figure 2 and Table A2. For the primary outcome, SLA resulted in significantly greater pain reduction during ambulation on POD7 compared with TLA (deltaVASP7_walk: −6.22 ± 2.60 vs. −5.06 ± 3.23, difference: −1.16, 95% CI: −1.95 to −0.37, *p* = 0.004, Cohen’s d = 0.39, representing a small effect). However, this difference did not reach the predefined MCID threshold of 1.3, indicating that it is not clinically meaningful despite having statistical significance. For coughing, the delta score showed a non-normal distribution: pain reduction was greater following SLA (deltaVASP7_cough: median −6 [−8 to −4] vs. −5 [−8 to −2], *p* = 0.005, r = 0.19), again representing a small effect size. For the co-primary outcome (deltaVASP2_walk), no significant difference was observed between groups (−2.65 ± 2.72 vs. −2.55 ± 3.56, *p* = 0.820). Pain at rest showed no significant difference between groups at any time point; however, pain at rest on postoperative day 7 (POD7) approached significance (*p* = 0.065).

The PSM analysis created 82 matched pairs, with excellent balance achieved across all baseline covariates (all standardized mean differences < 0.1). The pain-reducing advantages of SLA were amplified in the matched cohort (Table A3).

- deltaPOD7_walk: SLA showed greater pain reduction (−6.46 ± 2.28 vs. −4.80 ± 3.06, MD: −1.66, 95% CI: −2.49 to −0.83, *p* = 0.000, Cohen’s d = 0.61, medium effect)
- deltaPOD7_cough: SLA demonstrated superior pain reduction (−6 [−8 to −4] vs. −5 [−7 to −2], *p* = 0.003, r = 0.33)
- deltaPOD7_rest: The difference became statistically significant (−6.96 ± 2.30 vs. −5.95 ± 2.73, MD: −1.01, 95% CI: −1.78 to −0.24, *p* = 0.011, Cohen’s d = 0.40)

Notably, the effect size for the primary outcome increased from d = 0.39 in the unmatched analysis to d = 0.61 in the matched cohort, suggesting that baseline imbalances may have masked the true treatment effect. However, even this amplified difference remained below the MCID threshold.

### 3.3. Cosmetic Satisfaction Outcomes

**Figure 3:**
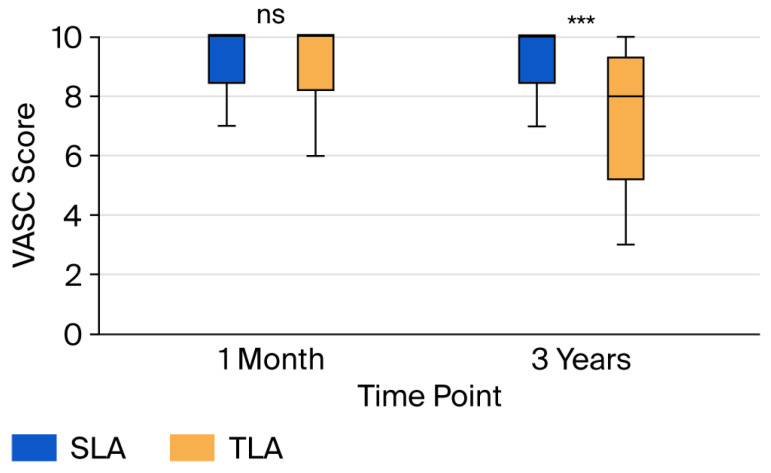
Comparison of cosmetic satisfaction at long-term follow-up. Box plots showing median (horizontal line), interquartile range (box), and 1.5 × IQR whiskers for Visual Analog Scale for Cosmesis (VASC, 0–10) at 1 month and 3 years postoperatively. The single-port laparoscopic appendectomy (SLA) group maintained high satisfaction scores at both time points, whereas the three-port laparoscopic appendectomy (TLA) group showed a decrease in satisfaction at 3 years. Outliers are represented as individual points, with statistical comparisons made using the Mann–Whitney U test. *** *p* < 0.001, ns: not significant (*p* > 0.05).

Long-term cosmetic satisfaction results are presented in Figure 3 and Table A2. VASC scores at both the 1-month and 3-year follow-ups showed non-normal distributions and are presented as median [IQR] values. At 1 month postoperatively, both groups showed high satisfaction, with no significant difference (SLA: 10 [9–10] vs. TLA: 10 [9–10], *p* = 0.177, Mann–Whitney U test, r = 0.09, representing a negligible effect). However, at 3 years postoperatively, the SLA group demonstrated significantly superior cosmetic satisfaction compared with the TLA group (VASC_3yrs: 10 [9–10] vs. 8 [6–9], *p* < 0.001, Mann–Whitney U test, r = 0.44, representing a medium-to-large effect). A separately calculated mean difference (9.39 ± 0.83 vs. 7.47 ± 1.98, 10.9 [1.48, 2.37], *p* = 0.00) exceeded the predefined MCID threshold of 1.5, indicating clinically meaningful improvement.

In the PSM analysis for cosmetic satisfaction, the cosmetic superiority of SLA was even more pronounced in the matched cohort (63 pairs with complete cosmetic follow-up, Table A3):

- VASC scores at 3 years remained significantly better for SLA patients (10 [9–10] vs. 8 [7–9], *p* < 0.001, Wilcoxon signed-rank test, r = 0.52, representing a large effect), confirming the robustness of the cosmetic benefit.
- The difference remained highly significant and clinically meaningful (9.56 ± 0.56 vs. 7.71 ± 1.63, MD: 1.84, 95% CI: 1.42 to 2.27, *p* = 0.000).

These PSM findings suggest that when baseline factors are balanced, the cosmetic advantages of SLA become apparent even at 1 month and are sustained over the long term.

### 3.4. Scar Perception Outcomes

**Figure 4:**
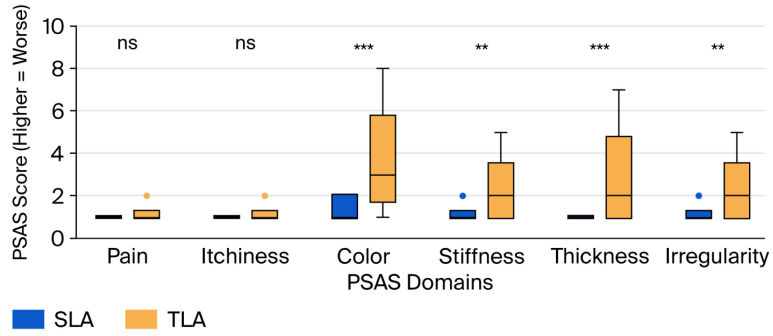
Comparison of scar perception at 3 year follow-up. Box plots showing median (horizontal line), interquartile range (box), and 1.5 × IQR whiskers for each domain of the Patient and Parental Scar Assessment Scale (PSAS). Each item is scored 0–10, with 10 representing the worst imaginable outcome. The three-port laparoscopic appendectomy (TLA) group showed significantly worse scores in the color, stiffness, thickness, and irregularity domains compared with the single-port laparoscopic appendectomy (SLA) group. Statistical comparison by the Mann–Whitney U test. ** *p* < 0.01, *** *p* < 0.001, ns: not significant (*p* > 0.05).

Detailed scar perception assessment at 3 years revealed significant differences across multiple domains (Figure 4, Table A2). All PSAS scores showed non-normal distributions and are reported as median [IQR] values. The TLA group demonstrated significantly worse perception, with medium effect sizes:

- Color: 1 [1–2] vs. 3 [2–5] (*p* = 0.000, r = 0.42, medium effect);
- Stiffness: 1 [1–1] vs. 2 [1–3] (*p* = 0.000, r = 0.31, medium effect);
- Thickness: 1 [1–1] vs. 2 [1–4] (*p* = 0.000, r = 0.38, medium effect);
- Irregularity: 1 [1–1] vs. 2 [1–3] (*p* = 0.000, r = 0.29, small-to-medium effect).

No significant differences were observed in the pain (1 [1–1] vs. 1 [1–1], *p* = 0.218, r = 0.08) or itchiness (1 [1–1] vs. 1 [1–1], *p* = 0.498, r = 0.04) domains, with both showing negligible effect sizes. The total PSAS score was significantly higher (worse) in the TLA group (7 [6–8] vs. 11 [8–17], *p* < 0.001, r = 0.45, medium-to-large effect).

The PSM analysis (63 matched pairs) confirmed all scar perception findings with consistent or larger effect sizes (Table A3):

- Color: 1 [1–1] vs. 3 [2–5] (*p* = 0.000, r = 0.48, medium effect size);
- Stiffness: 1 [1–1] vs. 2 [1–3] (*p* = 0.001, r = 0.36, medium effect size);
- Thickness: 1 [1–1] vs. 2 [1–4] (*p* = 0.000, r = 0.43, medium effect size);
- Irregularity: 1 [1–1] vs. 2 [1–3] (*p* = 0.002, r = 0.34, medium effect size);
- Total PSAS: 7 [6–8] vs. 11 [7–17] (*p* = 0.000, r = 0.51, large effect size).

### 3.5. Mediation Analysis

**Figure 5:**
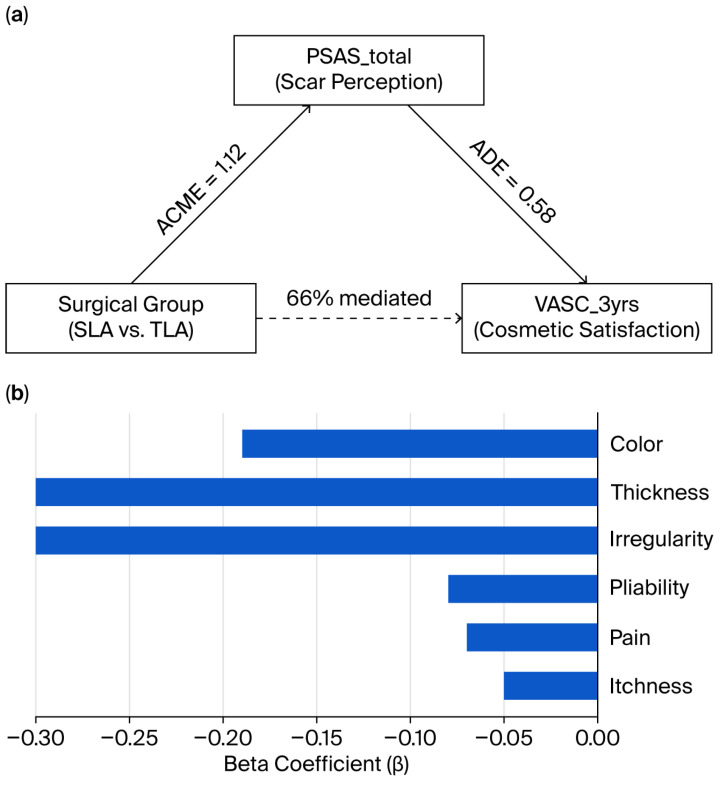
Mediation and regression analyses of scar perception and long-term cosmetic satisfaction. (**a**) Mediation model demonstrating that 66% of the total effect of the surgical group [single-port laparoscopic appendectomy (SLA) vs. three-port laparoscopic appendectomy (TLA)] on 3-year cosmetic satisfaction [Visual Analog Scale for Cosmesis (VASC_3yrs)] was mediated through overall scar perception [Patient or Observer Scar Assessment Scale (PSAS_total)]. The average causal mediation effect (ACME) was 1.12, and the direct effect (ADE) was 0.58. (**b**) Multivariable regression analysis showing standardized beta coefficients of PSAS subitems predicting VASC_3yrs. The sub-items for color, thickness, and irregularity were the most influential negative predictors.

Mediation analysis demonstrated that 66% of the total effect of surgical technique (SLA vs. TLA) on 3-year cosmetic satisfaction was mediated through overall scar perception (PSAS_total) (Figure 5, Table A4). The average causal mediation effect (ACME) was 1.12, with a direct impact (ADE) of 0.58 and a total effect of 1.70. Multivariable regression analysis identified color, thickness, and irregularity as the most influential negative predictors of cosmetic satisfaction, with standardized beta coefficients of −0.19, −0.30, and −0.30, respectively (Figure 5, Table A4). Pain and itchiness had minimal impact on long-term satisfaction (β = −0.07 and −0.05, respectively).

## 4. Discussion

This prospective comparative study provides comprehensive evidence of the practical benefits of single-port versus three-port laparoscopic appendectomy in pediatric patients. While SLA offers statistically significant improvements in postoperative pain reduction, these benefits fall short of clinical significance. Conversely, SLA demonstrates substantial and clinically meaningful advantages in long-term cosmetic satisfaction that persist at the 3-year follow-up.

Our study demonstrated that SLA resulted in statistically significant reductions in pain during ambulation (deltaVASP7_walk: −1.16, *p* = 0.004, d = 0.39) and coughing (median difference -1, *p* = 0.05, r = 0.19) at postoperative day 7. Despite statistical significance and small-to-medium effect sizes, these differences did not reach the predetermined MCID threshold of 1.3 points, indicating that these benefits are not clinically meaningful. The propensity score-matched analysis amplified these differences (walking: −1.66; coughing: r = 0.33), indicating robust findings that remain both unexplained by confounding and below the MCID threshold. These findings align with several recent studies questioning the pain benefits of SLA. Boo et al. compared transumbilical laparoscopic-assisted appendectomy with single-incision techniques in 142 children and found no significant differences in pain scores or analgesic requirements, concluding that the theoretical advantage of fewer incisions may not translate to meaningful pain reduction [26]. Similarly, a randomized controlled trial by Kang et al. (SCAR trial) involving 200 patients found a mean pain score difference in only 0.8 points between SLA and TLA, which falls below commonly accepted MCID thresholds [27].

In contrast, our results differ from studies reporting substantial pain benefits with SLA. Sozutek et al. reported mean pain scores of 3.93 versus 5.32 (difference: 1.39) in favor of SLA at 12 h postoperatively [10]. Several factors may explain these discrepancies. The umbilical fascial incision used in SLA (typically 2.0–2.5 cm) is larger than the individual port sites employed in TLA (5–10 mm each), potentially offsetting the benefit of fewer incisions [28]. Additionally, the technical complexity of SLA may lead to increased tissue manipulation and traction, particularly as the surgeon gains experience with the technique [29]. Studies showing larger pain differences often assessed pain only at rest or used non-validated scales [30]. Our activity-specific assessment revealed that pain differences emerged primarily during provocative activities (coughing or walking) rather than at rest, suggesting that functional pain assessment is critical for detecting differences between techniques [31]. Children have variable pain expression influenced by age, anxiety, and parental presence [32]. Studies with younger cohorts or different cultural contexts may report different pain outcomes independent of surgical technique [33]. Variations in perioperative analgesia, including regional blocks, wound infiltration, and systemic medications, can mask or amplify differences between surgical techniques [34]. Our standardized protocol (fentanyl and ketorolac) may have minimized baseline pain sufficiently that the incremental benefits of fewer incisions became negligible.

In marked contrast to pain outcomes, our study demonstrated clinically meaningful cosmetic benefits with SLA. The 1.93-point difference in VASC scores at 3 years substantially exceeded the MCID of 1.5, with PSM analysis confirming robustness (difference: 1.84, *p* = 0.0001). Notably, while the 1-month satisfaction scores were similar in the unmatched analysis, PSM revealed an early difference (r = 0.26, *p* = 0.020), suggesting that cosmetic benefits may manifest earlier than previously recognized when baseline factors are controlled. Our findings strongly support previous reports of cosmetic superiority with single-port techniques. A systematic review by Aly et al. analyzing 11 studies found consistently higher cosmetic satisfaction scores with SLA across different assessment tools and time points [35]. St. Peter et al. reported that 94% of parents preferred the appearance of the single-incision scar compared to 68% for multi-port scars at 6-month follow-up [36]. Zhang et al.’s long-term study found that cosmetic satisfaction scores continued to diverge between SLA and TLA groups from 6 months to 2 years, suggesting an increasing benefit over time [37]. Our findings align with recent pediatric studies emphasizing cosmetic outcomes. Qin et al. reported improved cosmetic outcomes with a transumbilical single-site double-port laparoscopic approach compared to standard three-port appendectomy [38]. Similarly, Phutane et al. found significantly better cosmetic acceptance with TULAA [39]. However, consistent with Abdullah et al., some studies show only modest cosmetic differences that may not reach statistical significance, indicating that while there is a trend toward improved cosmetic outcomes with single-incision methods, the extent and persistence of these benefits require long-term follow-up, as demonstrated in our 3-year assessment [40].

Some studies have reported minimal cosmetic differences. Ostlie et al. found no significant differences in scar satisfaction between SLA and TLA at 1-year follow-up using the Patient Scar Assessment Scale [41]. Gasior et al. reported that while parents initially preferred single-incision scars, this preference diminished by 6 months postoperatively [17]. There are key factors explaining these disparate findings. Our 3-year follow-up captured complete scar maturation, whereas studies with shorter follow-up (≤ 12 months) may miss late differences [42]. Pediatric scars undergo prolonged remodeling, with erythema and thickness evolving over 18 to 24 months [43]. Studies using surgeon-reported scales or non-validated measures may not capture patient-perceived differences [44]. Our use of both global satisfaction and domain-specific assessment provided a comprehensive evaluation of cosmetic outcomes. The cosmetic advantage of SLA depends on a meticulous umbilical placement and closure technique [45]. Centers with standardized protocols and experienced surgeons may achieve superior results compared with surgeons in the early stages of learning [46].

Our study provides novel mechanistic insights through detailed scar perception analysis and formal mediation testing. The finding that 66% of cosmetic satisfaction differences were mediated through scar perception establishes a clear causal pathway from surgical technique to patient satisfaction. Within PSAS domains, thickness, irregularity, and color emerged as primary drivers, while pain and itchiness contributed minimally to the overall assessment. These findings align with the fundamental principles of wound healing and scar formation. The single umbilical incision in SLA benefits from several anatomical advantages: (1) its placement within a natural skin depression minimizes visibility; (2) circular tension vectors around the umbilicus distribute stress evenly, reducing hypertrophy; (3) embryological scar tissue at the umbilicus may have different healing characteristics than normal skin [47].

In contrast, the peripheral incisions in TLA are subject to several disadvantages: (1) their placement across Langer’s lines increases tension and scar widening, (2) movement-related stress during recovery may promote hypertrophic changes, and (3) multiple scars increase the statistical probability of at least one poor cosmetic outcome [48]. Our findings, that thickness and irregularity dominate satisfaction, align with studies in plastic surgery, which show that raised, irregular scars are most bothersome to patients, while flat, well-contoured scars are often acceptable, regardless of color [49,50]. These have important clinical implications, suggesting that interventions targeting scar thickness (such as silicone sheets and compression) and contour (including massage and taping) may be particularly beneficial for TLA patients [51].

Based on this study, especially the finding that pain differences did not reach the MCID threshold, has important implications for surgical decision-making. The effect sizes observed (d = 0.39–0.61 for pain reduction) suggest that while SLA does provide some benefit, the magnitude may not justify choosing this technique solely for pain management. Clinicians should not recommend SLA primarily for pain reduction benefits. Instead, the decision should be based on other factors, particularly the family’s prioritization of long-term cosmetic outcomes (r = 0.44–0.52, medium-to-large effect), the surgeon’s experience, and the potential for complications, if adolescent patients have strong body image concerns, and whether patients have a history of hypertrophic scarring [52].

However, essential limitations merit consideration. Although propensity score matching was performed and demonstrated increased effect sizes in the matched cohort, the lack of randomization remains a limitation. The choice of surgical approach was made by the operating surgeon based on their experience and preference, following a discussion with the family. This non-randomized allocation represents a significant limitation of our study, as unmeasured confounders related to surgeon preference or patient factors may have influenced outcomes despite our propensity score-matching approach.

Our single-center design with experienced surgeons (>100 cases of both SLA and TLA) may limit external applicability. Centers with surgeons in earlier stages of learning may experience different outcomes, particularly longer operative times and potentially higher complication rates with SLA due to its technical complexity. Multi-center studies including surgeons with varying experience levels are needed to confirm generalizability.

Our study’s relatively narrow inclusion criteria (uncomplicated appendicitis, age ≤15 years, no previous abdominal surgery) may limit the generalizability of its results. Outcomes may differ in complicated appendicitis cases, older adolescents, or patients with previous abdominal surgery. The subjective nature of pain and cosmetic assessment, even when using validated instruments, remains inherently variable. The 3-year follow-up period, while comprehensive for scar assessment, cannot capture very long-term outcomes in adulthood.

Several research priorities emerge from our findings. Randomized controlled trials with standardized techniques and long-term follow-up, as well as multi-center studies to enhance generalizability, would provide definitive evidence. The development of pediatric-specific cosmetic outcome measures that incorporate developmental considerations would enhance assessment and treatment. Investigation of scar prevention strategies (technique modifications and adjuvant therapies) could benefit both SLA and TLA patients. A cost-effectiveness analysis that incorporates long-term satisfaction and potential revision procedures would inform health policy. Additionally, studies examining outcomes in complicated appendicitis, as well as the evaluation of new single-port techniques and instruments, would be valuable. Ultimately, exploring the determinants of patient and parent preferences could inform the development of shared decision-making tools.

## 5. Conclusions

This prospective comparative study demonstrates that single-port laparoscopic appendectomy provides statistically significant but clinically marginal benefits in early postoperative pain reduction compared with three-port laparoscopic appendectomy in pediatric patients. However, SLA does offer substantial and clinically meaningful advantages in long-term cosmetic satisfaction, which is primarily mediated through improved scar characteristics, including reduced thickness, irregularity, and color mismatch. These findings support SLA as a valuable option for pediatric appendectomy, particularly when families prioritize long-term cosmetic outcomes.

## Figures and Tables

**Table 1:** Baseline characteristics of single-port vs. three-port laparoscopic appendectomy groups.

Variable	SLA (*n* = 127)	TLA (*n* = 111)	MD or OR [95% CI], or U	*p*-Value
Age, year	10.1 ± 3.0	9.8 ± 3.1	0.34 [−0.46, 1.14]	0.407
Sex, male (%)	53 (41.7)	40 (36.0)	1.27 [0.75, 2.15]	0.425
Weight, kg	39.3 ± 14.7	38.3 ± 14.9	0.93 [−2.83, 4.69]	0.629
BMI, kg/m^2^ *	18.3 [15.9–20.5]	17.8 [15.8–19.9]	5524	0.871
WBC, ×10^3^	14.2 ± 5.2	14.5 ± 4.9	−0.31 [−1.59, 0.97]	0.632
Neutrophil, % *	80.9 [73.7–86.2]	82.2 [73.6–87.3]	6156	0.397
CRP *	7.9 [1.6–31.0]	6.9 [1.6–25.0]	5892	0.770
Operation time, min *	35 [30–50]	40 [30–55]	6432	0.360
Postoperative days *	2 [2–2]	2 [2–2]	6987	0.717
Wound seroma	3 (2.4%)	3 (2.7%)	0.87 [0.17, 4.41]	0.871
Postoperative ileus	3 (2.4%)	2 (1.8%)	1.32 [0.22, 8.04]	1.000

SLA: single-port laparoscopic appendectomy; TLA: three-port laparoscopic appendectomy; MD: mean difference for normally distributed variables; OR: odds ratio for categorical variables; CI: confidence interval; U: Mann–Whitney U statistic values for non-normally distributed variables; BMI: body mass index; * non-normally distributed variables presented as median [IQR]; WBC: white blood cells; CRP: C-reactive protein.

## Data Availability

The data presented in this study are available from the corresponding author on request.

## Abbreviations

The following abbreviations are used in this manuscript:

SLA	Single-port laparoscopic appendectomy
TLA	Three-port laparoscopic appendectomy
POD	Postoperative days
VASP	Visual Analog Scale for Pain
VASC	Visual Analog Scale for Cosmesis
PSAS	Patient and Parental Scar Assessment Scale
PSM	Propensity score matching
MCID	Minimal clinically important difference
ACME	Average causal mediation effect
ADE	Average direct effect

## Appendix A

**Table A1:** Six-item validated Patient and Parental Scar Assessment Scale.

Patient and Parental Scar Assessment Scale			
	No complaint	1 2 3 4 5 6 7 8 9 10	Worst imaginable
Is the scar painful?			
Is the scar itching?			
	As normal skin	1 2 3 4 5 6 7 8 9 10	Very different
Is the color of the scar different?			
Is the scar stiffer?			
Is the thickness of the scar different?			
Is the scar irregular?			

**Table A2:** Outcomes of pain reduction, global cosmetic satisfaction, and scar perception. Actual values of pain over time, differences in pain between preoperative and postoperative values, cosmetic satisfaction score at 1 month and 3 years after surgery, and 6-item scar perception scores were summarized between single and three-port laparoscopic appendectomy.

Variable	SLA (Mean, SD or Median, IQR)	SLA (*n*)	TLA (Mean, SD or Median, IQR)	TLA (*n*)	MD [95% CI] or U	*p*-Value	Effect Size
VASPpreop_rest	6.87 ± 2.42	121	6.46 ± 2.57	101	0.41 [−0.25, 1.07]	0.221	d = 0.16
VASP1_rest	5.39 ± 2.56	121	5.31 ± 2.60	101	0.09 [−0.59, 0.77]	0.803	d = 0.03
VASP2_rest	3.13 ± 2.10	121	2.95 ± 1.99	101	0.18 [−0.36, 0.72]	0.509	d = 0.09
VASP7_rest *	0 [0–0]	118	0 [0–1]	99	5525	0.321	r = 0.07
VASPpreop_cough	6.90 ± 2.47	121	6.32 ± 2.68	101	0.58 [−0.10, 1.27]	0.094	d = 0.22
VASP1_cough	7.09 ± 2.06	121	6.86 ± 2.14	101	0.23 [−0.33, 0.78]	0.419	d = 0.11
VASP2_cough	5.07 ± 1.92	121	5.00 ± 2.00	101	0.07 [−0.45, 0.59]	0.803	d = 0.04
VASP7_cough *	0 [0–2]	118	1 [0–2]	99	5291	0.080	r = 0.12
VASPpreop_walk	6.94 ± 2.50	121	6.54 ± 2.65	101	0.40 [−0.28, 1.08]	0.253	d = 0.15
VASP1_walk	6.18 ± 2.26	121	6.10 ± 2.11	101	0.08 [−0.49, 0.66]	0.781	d = 0.04
VASP2_walk	4.30 ± 1.99	121	3.99 ± 1.98	101	0.31 [−0.22, 0.83]	0.250	d = 0.16
VASP7_walk *	0 [0–1]	118	1 [0–2]	99	4877	0.002	r = 0.21
deltaVASP1_rest	−1.48 ± 3.35	121	−1.15 ± 3.34	101	−0.33 [−1.21, 0.56]	0.468	d = 0.10
deltaVASP2_rest	−3.74 ± 3.10	121	−3.50 ± 3.25	101	−0.24 [−1.08, 0.60]	0.577	d = 0.08
deltaVASP7_rest	−6.53 ± 2.48	118	−5.86 ± 2.82	99	−0.68 [−1.39, 0.04]	0.065	d = 0.25
deltaVASP1_cough	0.19 ± 2.77	121	0.54 ± 3.00	101	−0.36 [−1.12, 0.41]	0.361	d = 0.12
deltaVASP2_cough	−1.84 ± 2.81	121	−1.32 ± 3.30	101	−0.53 [−1.34, 0.29]	0.207	d = 0.17
deltaVASP7_cough *	−6 [−8 to −4]	118	−5 [−8 to −2]	99	4892	0.005	r = 0.19
deltaVASP1_walk	−0.76 ± 2.88	121	−0.45 ± 3.03	101	−0.32 [−1.10, 0.46]	0.427	d = 0.11
deltaVASP2_walk	−2.65 ± 2.72	121	−2.55 ± 3.56	101	−0.10 [−0.95, 0.75]	0.820	d = 0.03
deltaVASP7_walk	−6.22 ± 2.60	118	−5.06 ± 3.23	99	−1.16 [−1.95, −0.37]	0.004	d = 0.39
VASC_1month *	10 [9–10]	111	10 [9–10]	97	5164	0.177	r = 0.09
VASC_3yrs *	10 [9–10]	102	8 [6–9]	90	2835	0.000	r = 0.44
PSAS_pain *	1 [1–1]	102	1 [1–1]	90	4419	0.218	r = 0.09
PSAS_itchiness *	1 [1–1]	102	1 [1–1]	90	4545	0.498	r = 0.05
PSAS_color *	1 [1–2]	102	3 [1–5]	90	2548	0.000	r = 0.42
PSAS_stiffness *	1 [1–1]	102	2 [1–3]	90	3244	0.000	r = 0.31
PSAS_thickness *	1 [1–1]	102	2 [1–4]	90	2870	0.000	r = 0.38
PSAS_irregularity *	1 [1–1]	102	2 [1–3]	90	3397	0.000	r = 0.29
PSAS_total *	7 [6–8]	102	11 [8–17]	90	2403	0.000	r = 0.45

SLA: single-port laparoscopic appendectomy; TLA: three-port laparoscopic appendectomy; MD: mean difference for normally distributed variables; CI: confidence interval: U: Mann–Whitney U statistic value for non-normally distributed variables; VASP: visual analog scale for pain; VASC: visual analog scale for cosmesis; PSAS: patient and parental scar assessment scale; * non-normally distributed variables presented as median [IQR]. Effect sizes: d = Cohen’s d for parametric tests; r = correlation coefficient (Z/√N) for non-parametric tests.

**Table A3:** Propensity score matching outcomes of pain reduction, global cosmetic satisfaction, and scar perception. Actual values of pain over time, differences in pain between preoperative and postoperative values, cosmetic satisfaction score at 1 month and 3 years after surgery, and 6-item scar perception scores were summarized between single and three-port laparoscopic appendectomy.

Variable	SLA (Mean, SD or Median, IQR)	SLA (*n*)	TLA (Mean, SD or Median, IQR)	TLA (*n*)	MD [95% CI] or W	*p*-Value	Effect Size
VASPpreop_rest	7.26 ± 2.34	82	6.37 ± 2.54	82	0.89 [0.14, 1.64]	0.020	d = 0.36
VASP1_rest	5.01 ± 2.47	82	5.02 ± 2.62	82	−0.01 [−0.79, 0.77]	0.975	d = 0.02
VASP2_rest	3.23 ± 2.27	82	3.07 ± 2.02	82	0.16 [−0.50, 0.82]	0.637	d = 0.08
VASP7_rest *	0 [0–0]	82	0 [0–1]	82	1653	0.378	r = 0.10
VASPpreop_cough	6.95 ± 2.45	82	6.20 ± 2.63	82	0.76 [−0.02, 1.53]	0.058	d = 0.29
VASP1_cough	6.76 ± 1.97	82	6.62 ± 2.14	82	0.13 [−0.50, 0.76]	0.676	d = 0.10
VASP2_cough	4.67 ± 1.85	82	4.95 ± 2.14	82	−0.28 [−0.89 to 0.33]	0.370	d = 0.05
VASP7_cough *	0 [0–1]	82	1 [0–2]	82	1456	0.066	r = 0.20
VASPpreop_walk	7.10 ± 2.42	82	6.27 ± 2.63	82	0.83 [0.06, 1.60]	0.037	d = 0.33
VASP1_walk	5.54 ± 2.29	82	5.89 ± 2.17	82	−0.35 [−1.04, 0.33]	0.312	d = 0.04
VASP2_walk	3.87 ± 1.88	82	4.09 ± 1.99	82	−0.22 [−0.81, 0.37)	0.468	d = 0.16
VASP7_walk *	0 [0–1]	82	1 [0–2]	82	1089	0.000	r = 0.43
deltaVASP1_rest	−2.24 ± 3.64	82	−1.34 ± 3.35	82	−0.90 [−1.97, 0.17]	0.100	d = 0.10
deltaVASP2_rest	−4.02 ± 3.40	82	−3.29 ± 3.18	82	−0.73 [−1.74, 0.27]	0.156	d = 0.08
deltaVASP7_rest	−6.96 ± 2.30	82	−5.95 ± 2.73	82	−1.01 [−1.78, −0.24]	0.011	d = 0.40
deltaVASP1_cough	−0.20 ± 2.73	82	0.43 ± 3.10	82	−0.62 [−1.52, 0.27]	0.174	d = 0.12
deltaVASP2_cough	−2.28 ± 2.72	82	−1.24 ± 3.39	82	−1.04 [−1.98, −0.10]	0.032	d = 0.17
deltaVASP7_cough *	−6 [−8 to −4]	82	−5 [−7 to −2]	82	1234	0.003	r = 0.33
deltaVASP1_walk	−1.56 ± 2.87	82	−0.38 ± 3.08	82	−1.18 [−2.09, −0.27]	0.011	d = 0.12
deltaVASP2_walk	−3.23 ± 2.77	82	−2.18 ± 3.46	82	−1.05 [−2.01, −0.09)	0.033	d = 0.04
deltaVASP7_walk	−6.46 ± 2.28	82	−4.80 ± 3.06	82	−1.66 [−2.49, −0.83]	0.000	d = 0.61
VASC_1month *	10 [9–10]	63	9 [8–10]	63	892	0.020	r = 0.26
VASC_3yrs *	10 [9–10]	63	8 [7–9]	63	378	0.000	r = 0.52
PSAS_pain *	1 [1–1]	63	1 [1–1]	63	1024	0.057	r = 0.21
PSAS_itchiness *	1 [1–1]	63	1 [1–1]	63	126	1.000	r = 0.00
PSAS_color *	1 [1–1]	63	3 [2–5]	63	456	0.000	r = 0.48
PSAS_stiffness *	1 [1–1]	63	2 [1–3]	63	687	0.001	r = 0.36
PSAS_thickness *	1 [1–1]	63	2 [1–4]	63	523	0.000	r = 0.43
PSAS_irregularity *	1 [1–1]	63	2 [1–3]	63	712	0.002	r = 0.34
PSAS_total *	7 [6–8]	63	11 [7–17]	63	398	0.000	r = 0.51

SLA: single-port laparoscopic appendectomy; TLA: three-port laparoscopic appendectomy; MD: mean difference for normally distributed variables; CI: confidence interval; W: Wilcoxon signed-rank statistic value for non-normally distributed variables; VASP: visual analog scale for pain; VASC: visual analog scale for cosmesis; PSAS: patient and parental scar assessment scale; * non-normally distributed variables presented as median [IQR]. Effect sizes: d = Cohen’s d for parametric tests; r = correlation coefficient (Z/√N) for non-parametric tests.

**Table A4:** Mediation and regression analysis. (a) Mediation analysis results showing the indirect effect (ACME), direct effect (ADE), total effect, and the proportion of the total impact mediated by Patient and Parental Scar Assessment Scale (PSAS_total). (b) Multivariable linear regression coefficients (β) of each PSAS subitem predicting 3-year global cosmetic satisfaction (Visual Analog Scale for Cosmesis at 3 years after surgery).

(a)	
Effect	Estimate
ACME (indirect)	1.12
ADE (direct)	0.58
Total Effect	1.70
Proportion Mediated	0.66
(b)	
PSAS Subitem	Beta Coefficient (β) Predicting VASC_3yrs
Color	−0.19
Thickness	−0.30
Irregularity	−0.30
Pliability	−0.08
Pain	−0.07
Itchiness	−0.05

ACME: Average causal mediation effect; ADE: Average direct effect; PSAS: patient and parental scar assessment scale; VASC: visual analog scale for cosmetic satisfaction.
